# Effect of Chemical Aggressive Media on the Flexural Properties of Cured-In-Place Pipes Supported by Microstructure Observation and Acoustic Emission

**DOI:** 10.3390/ma13143051

**Published:** 2020-07-08

**Authors:** Jakub Hodul, Jana Majerová, Rostislav Drochytka, Richard Dvořák, Libor Topolář, Luboš Pazdera

**Affiliations:** 1 Faculty of Civil Engineering, Institute of Technology of Building Materials and Components, Brno University of Technology, Veveri 95, 602 00 Brno, Czech Republic; majerova.j@fce.vutbr.cz (J.M.); drochytka.r@fce.vutbr.cz (R.D.); 2 Faculty of Civil Engineering, Institute of Physics, Brno University of Technology, Veveri 95, 602 00 Brno, Czech Republic; dvorak.r1@fce.vutbr.cz (R.D.); Libor.Topolar@vut.cz (L.T.); pazdera.l@fce.vutbr.cz (L.P.)

**Keywords:** cured-in-place pipe (CIPP), chemical stress, microstructure, acoustic emission, flexural properties, thermal exposure

## Abstract

The cured-in-place pipe (CIPP) method is currently the most frequently used approach for the renovation of piping without digging; this technology is suitable for pipes made from all types of material. The authors of this paper examined how chemical substances and increased temperature change samples of CIPP with vinyl-ester resin taken from a simulated installation. Changes were observed at several levels: visually via a digital optical microscope, through changes of short-term bending properties and by observation of the activity of the sample structure by means of acoustic emission (AE). Interdependencies among the observed parameters were examined, specifically, the cumulative number of hits (cnt)/deflection and flexural properties/mechanic wave velocity. The test results prove that after three weeks of immersion in a simulated aggressive environment that mirrors what may happen to CIPP in real conditions, short-term mechanical properties change. This is also proven by the results of the AE measurements. For clarity, the results include images from a digital optical microscope. In addition, this paper proves that CIPP samples have good resistance to the action of organic and inorganic acids and to increased temperatures. After three weeks of exposure to a temperature of 100 °C the CIPP flexural properties of the samples had even improved.

## 1. Introduction

Sewage pipelines are a crucial part of city infrastructure; once they are constructed, they are expected to remain in use for 20 years [1]. Cured-in-place pipe (CIPP) is a method of renovating piping without digging that was invented in 1971. In 1975, a request for a patent for this technology was submitted; it was obtained in 1977 [2,3]. After the patent was released in 1994, this technology spread all over the world and was innovated [4]. It became popular due to its advantages, such as rapid installation, less restriction of traffic and life around the work site and less required manipulation of the area compared to digging work. A further unambiguous advantage is the applicability of this method in town centers and densely built-up areas. This is shown by the fact that the CIPP method is used for most renovation works that are done without digging [5,6]. Some publications state that the CIPP method is more ecological because less water escapes from the renovated pipes, compared to other materials used [7]. In typical CIPP applications, a lining tube saturated with a styrene-based thermosetting resin is installed into the pipe requiring rehabilitation. Subsequent curing with a heat source results in a pipe within a pipe [8].

The principle of the method is based on the saturation of a sleeve (made from woven or unwoven textile) with thermoset resin; the saturated sleeve then hardens in the place. Thus, the result of the renovation is the creation of a composite pipe inside the renovated pipe. The new pipe reduces the diameter of the original pipe; however, the hydraulic parameters are improved.

Pipes are subjected to numerous negative loads and influences such as traffic, changes in subsoil, seismic activity or chemical action. These external factors can damage renovated pipes [9].

The use of resins is based on their properties, for instance, good adhesion to renovated pipes, excellent chemical resistance (in particular vinyl ester-based resins), variability of shapes and good mechanical properties.

The most frequently used resin bases are polyester, vinyl-ester and epoxide. Polyester and vinyl ester resins (VESs) have similar chemical bases; the main difference is the location of the reactive groups at the end of the molecular chain. This makes the resistance of VESs greater than that of polyester [9]. It was found that the polyester reaction is slightly more sensitive to temperature change than the vinyl ester reaction. Vinyl ester resins have terminal carbon–carbon double bonds, and hence, are more reactive than unsaturated polyester resins, which contain internal carbon–carbon double bonds [10]. It was proved by Ribeiro et al. [11] that during long-term immersion in a sulfuric acid solution, the flexural strength of epoxy polymer concrete decreased to approximately four times less than that of polyester polymer concrete. Vinyl ester resin shows better chemical stability than polyester, which is more susceptible to hydrolysis, especially when not fully cured and where the extraction of low molecular weight polyester species is enhanced. Polyester isophthalic achieves a lower degree of conversion than does vinyl ester [12,13]. Regarding resistance to chemicals, existing results state that polyester resins have the lowest resistance, epoxide resins are in the middle and VESs have the highest resistance [14,15]. For these reasons, the authors of this paper decided to focus on the vinyl-ester matrix of the composite material.

Each instance of CIPP renovation without digging is specific and unique; therefore, the properties of the resulting composite material depend on the conditions of installation. The design and calculation stage of a renovation estimates a 50-year life-span of the renovation. So far, few studies have compared the short-term flexural properties at the time of installation with those after several years of service. The available results imply that, over time, the values of the short-term flexural properties increase [16].

Suzuky et al. [17] investigated the various types of failure of a model polyester-matrix composite with one layer of quartz bers. They performed an analysis using acoustic signals due to ber breakage and matrix debonding by incorporating a viscoelastic matrix model. Farhidzadeh et al. [18] used the acoustic emission (AE) technique for the investigation of two CIPP damage mechanisms—delamination between pipeline and liner and incipient failure of the liner. The AE features were also studied by Farhidzadeh et al. [19] to detect their appropriateness for CIPP failure detection, to classify fracture from debonding and to highlight liner failure at quasi-static and dynamic loading.

AE testing is a nondestructive testing method. It has the advantage of only detecting active failures in the material and not geometrical imperfections. The term AE denotes the generation of transient elastic waves caused by the sudden redistribution of tension in the material [20]. When the structure of the observed test body, construction or material is exposed to the external stimulus, for example, a change of pressure, load, temperature or structure, localized sources trigger the release of energy in the form of tension waves. These waves then spread from the source through the material structure to the surface, where they can be detected via piezoelectric sensors [21].

Systems based on AE are usually only capable of providing qualitative measurements of the number of active defects contained in the structure. To obtain quantitative results on the size, depth and the overall condition of the sample, the use of other methods of nondestructive testing, either simultaneously or subsequently, is advisable. Another disadvantage of the AE method is its sensitivity towards the disturbing influence of the environment, which may bias the signal [22].

AEs mainly arise during the initiation and growth of cracks, gliding, slip or dislocation movement, or during phase changes within the structure of the tested material. AEs are produced solely when the material is subjected to strain; hence, inner tension is accumulated. Depending on the extent of the external tension and the properties of the material, the object will either return to its original dimensions or remain permanently deformed after the external tension is released. These two conditions are known as elastic or plastic deformation [23].

AEs are active when loaded material is subjected to plastic deformation. On a microscopic level, when plastic deformation occurs, atomic layers move around due to the movement of the dislocations. These atomic deformations release energy in the form of elastic waves, which can be considered a naturally generated sound or ultrasound that propagates through the object [21,23]. If there are cracks in the material, the level of tension in front of the tip of the crack can be sevenfold higher than that in the surrounding area.

The amount of energy released during AE and the amplitude is related to the size and velocity of the event generated by the acoustic-emitting source. Energy and AE are proportional to the velocity of crack propagation and the expanse of the area created on the surface of those cracks. Large changes in cracks, i.e., rapid propagation of cracks, cause larger signals of AE compared to cracks that propagate slowly for the same distance. The detection and transformation of these elastic waves into electric signals are the basis of the AE method. The analysis of these signals provides valuable information about the origin and importance of discontinuities in the observed material [24].

Any Structural Health Monitoring (SHM) system relies on preprocessed data, and on features extrapolated from them. Mel-Frequency Cepstral Coefficients (MFCCs) have been proven to be effective in damage detection, relying on the cepstrum of the recorded structural response [25]. The viability of using CCs also for damage estimation, and the notion that the concept of SHM based on MFCC-like features can still be improved, were discussed by Ferraris et al. [26].

This paper is based on existing studies; it examines the effect of chemical and thermal loading on the short-term mechanical properties of composite materials, and researches AE during sample loading. Changes in the microstructures were observed using an optical microscope.

## 2. Materials and Methods

### 2.1. Composition of CIPP Sample

The samples of CIPP that were measured were made from VES with a polypropylene (PP) layer, and were labelled PP/ Ethylene-Propylene Diene Monomer rubber (EPDM). This epoxy VES was based on bisphenol-A epoxy resin and has become the industry standard due to its wide range of end-use applications. DERAKANE 411-45 resin exhibits excellent mechanical properties over an entire service temperature range down to −50 °C, and has very high damage resistance. The resin contains 50% by volume of styrene and has a viscosity of 400–600 mPa·s at a temperature of 23 °C. Sokolowska states in her experimental study [27] that the excellent durability of polymer composites is mainly a consequence of the presence of a significant amount of polymer, which enables tighter matrices to be obtained. PP is modified with special EPDM rubber, which ensures the required impact resistance of the inner membrane. The lining of all the samples was made from the polyester (PES) and the host pipe was made from earthenware. The samples were hardened in hot water at a temperature of approximately 86 °C. The dimensions of the samples and the span of the supports were in accordance with the relevant standard, i.e., EN ISO 11296-4. In total, three sets of samples made up of five pieces each were prepared for the observation of the relevant parameters. The samples were labelled as CIPP VES1, CIPP VES2 and CIPP VES3, and each of the sets was placed in a different aggressive environment.

### 2.2. Monitoring of Chemical Resistance

For the purpose of the experiment, five aggressive environments were selected. Three of these were aggressive liquid solutions and two were drying rooms with an increased temperature. Chemical loading was simulated using three lab 300 mm clear glass desiccators with a porcelain plate filled with the chemical solutions. The first desiccator contained a 15% solution of acetic acid (CH_3_COOH), the second contained a 10% solution of ammonium hydroxide (NH_4_OH) and the third contained a 20% solution of hydrochloride acid (HCl). The specific types of aggressive environments were based on the data in the technical data sheet of the resin used, where the increase or loss of weight after 30 days of immersion in chemicals at a temperature of 22 °C is stated for the determined chemical resistance. The chemical resistance of the resin to 30% sulfuric acid, 10% acetic acid, 10% sodium hydroxide and 10% sodium chloride was tested by the manufacturer. Therefore, other concentrations or other types of hydroxides and acids were used in the experiment, which should be more aggressive. The resin hardens at a temperature of 80 °C; at temperatures above 100 °C, it degrades. Furthermore, these aggressive conditions were also chosen with regard to the use of CIPP pipes for the rehabilitation of sewage and industrial waste pipes, in which wastewater flows with a low pH and both inorganic and organic carboxylic acids are present. Higher concentrations of chemicals and higher temperatures were chosen to accelerate the degradation of the samples.

The height of the surface of the solution above the samples was 20 mm, and the desiccators were placed at laboratory temperature and humidity (23 °C and 60% R.H.). Thermal load was simulated by means of laboratory dryers with forced ventilation. The first dryer was set at 100 °C and the second at 130 °C. The samples were gradually taken from their different environments, and their properties at a given chemical and thermal resistance were tested every seven days, i.e., the first sample’s flexural properties were tested after seven days and the last one’s after 28 days. Their microstructure was studied before they were placed in the aggressive environment (reference samples) and 28 days after they had been placed in the aggressive environment, when the effect of the action of the specific aggressive solution or high temperature on their microstructure was evaluated.

### 2.3. Determination of Flexural Properties

**Figure 1:**
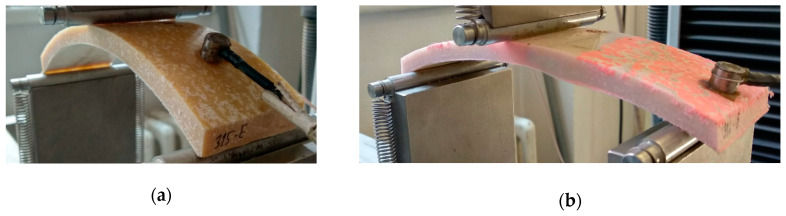
Testing the flexural properties: (**a**) Sample CIPP VES2 after two weeks at a temperature of 130 °C; (**b**) Sample CIPP VES3 after two weeks of immersion in NH_4_OH.

Flexural properties are among the most important basic mechanical properties of CIPP tubes. To determine flexural properties, three parameters were measured, namely the short-term flexural elasticity modulus (E_0_), flexural strength at break (σ_fb_) and flexural deformation at break (ε_fb_). These values were determined in accordance with EN ISO 11296-4. The geometrical dimensions of the samples used for the testing, including the radius of curvature, are stated in Table 1. Testing was carried out using a universal testing machine (Testometric M350-20CT) with a range of 20 kN, and the loading rate was 10 mm/min. Samples loaded for the three-point flexural test are depicted in Figure 1a,b. This test examined if the flexural properties of the CIPP samples decreased after being exposed to the aggressive environments described in Section 2.2.

### 2.4. Acoustic Emission

**Figure 2:**
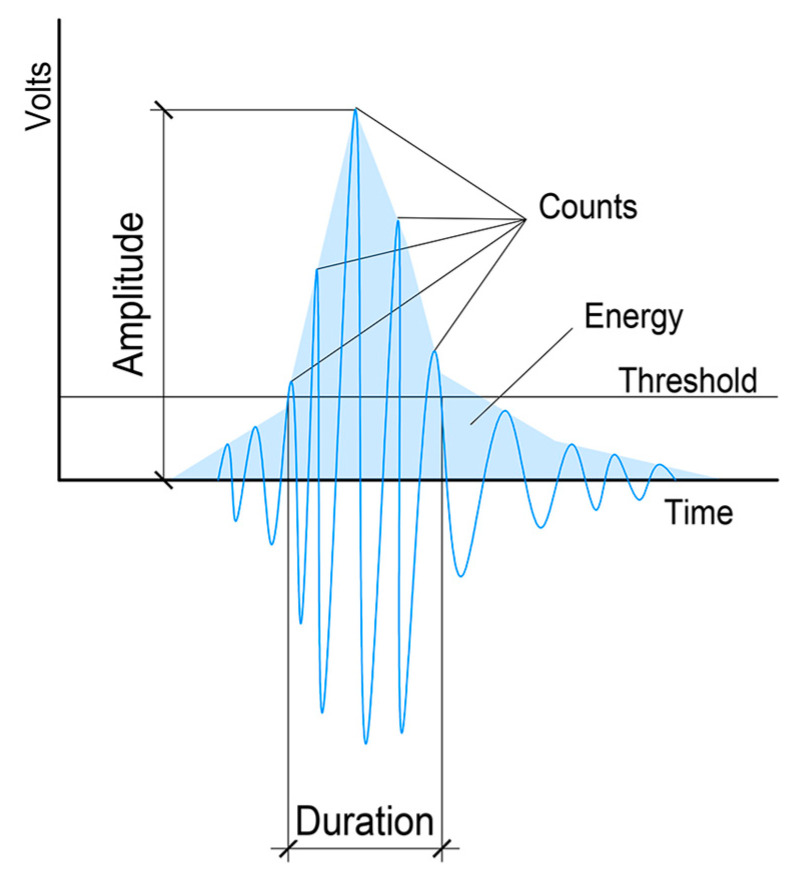
Example of an AE subsignal and its monitored parameters: number of threshold level overshoots, AE signal energy, signal length above threshold level and maximum signal amplitude.

The energy of the generated wave was detected and decoded using specialized equipment that is capable of automatically evaluating and storing useful signal information. This specialized equipment can be seen as a measurement chain [28]. The first link of the chain is the sensors. These are usually piezoelectric sensors, which are mainly high frequency acceleration sensors (in the order of MHz). Amplification using a preamp is usually in the range of 36 dB to 60 dB. Analyses can be carried out either during or after measurements; the latter is more frequently the case. The evaluation of the AE signals distinguishes individual signals (Figure 2). Cracking AE is a qualitative description of a discrete signal related to individual emission events in the material. Here, it is possible to unambiguously distinguish individual events in the signal. Continuous AE is a qualitative description of a constant signal produced by signals overlapping in time [29].

The cracking signal of an AE can be described using the parameters of AE as the amplitude of the signal—maximal value, the energy of the AE—integrated signal or intensity of rectified potential, time duration—time from first exceeding the threshold value until the last time it exceeds the threshold, number of exceeding threshold value—total number of times it exceeds the threshold value, and average frequency—average frequency of the event.

AE signals can be generated using the Pen-Test method (Hsu-Nielsen’s source of AE), whereby breaking a piece of lead with certain parameters (hardness 2H, diameter 0.3 or 0.5 mm) generates waves similar to those emitted by a source of AE. The principle is based on putting a pencil with a special calibrated ring onto a surface and breaking 3 mm long lead against the surface of the tested body. In this way the apparatus is calibrated for measurement, in particular for the possible localization of the position of the source of AE. This source of AE can also be used for the approximate determination of the velocity of the propagation of waves [30,31,32].

#### Description of the Experiment—Set for Measurement of Activity of AE

**Figure 3:**
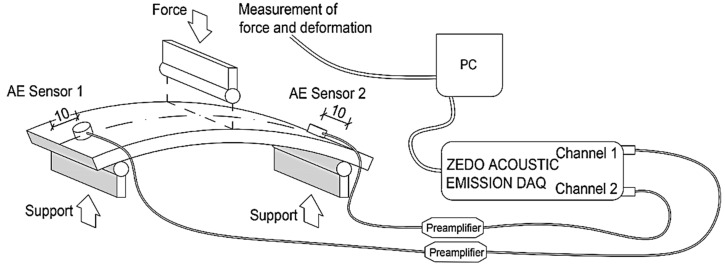
Sample attachment scheme and location of AE sensors.

Activity of AE was observed using a DAKEL-ZEDO system (DAKEL, Hořovice, Czech Republic), which is a modular system for measuring AE that can be used throughout the entire spectrum of the application of this diagnostic method, for example, for the detection and localization of the formation and development of the failure of materials. In order to measure this, two channel unit ZEDO-AE were used. These are intended for the processing of signals of AE. Observations of activity of AE were made at the same time as the determination of flexural properties; therefore, the samples used are the ones that are depicted undergoing the tree-point flexural loading, as well as the ones used for the localization of sensors (Figure 3). The sensors were attached on the specimen surface with beeswax. In order to measure the mechanic wave velocity and cumulative hits (cnt) as accurately as possible, the sensors were located 10 mm from the edges of the samples, which had a length of 150 to 160 mm and a width of 50 mm, as required by the relevant standard, i.e., EN ISO 11296-4. To minimize side effects, a so-called guard sensor was mounted on the test equipment.

The monitoring and recording of the acoustic wave were carried out using a MIDI (IDK 09, DAKEL, Hořovice, Czech Republic) piezoelectric sensor with a diameter of 6 mm and a height of 6.3 mm. The electric signal from this sensor was amplified using a 34 dB preamp (DAKEL, Hořovice, Czech Republic). The measurements and basic evaluation were carried out using ZDaemon software.

### 2.5. Microstucture Observation

To examine the microstructure of the VES-based CIPP samples, a Keyence VHX-950F digital optical microscope (DAKEL, Hořovice, Czech Republic) was used. This microscope has a maximal magnification of 200×, which corresponds to 67.7× real magnification. Digital microscopes make it possible to observe objects in the field of vision, and to save the images and evaluate the measured data. The advantages of this apparatus are its automatic focus, the fact that its depth of field is 20 times larger than that of standard optical microscopes, its high dynamic range (DHR+), which makes it possible to even observe low contrast and shiny objects, its optimization of light to produce high quality images, its ability to measure depth using the Depth from Defocus (DFD) method and the ability to change the angle of observation. The test specimens selected for this experiment were cuts of CIPPs with approximate dimensions of 50 × 50 mm. Observation of each of these materials was carried out on five test samples from both sides at a magnification of 20× (a real magnification of 6.4×). To accentuate the pores for better observations (size, distribution), the samples were impregnated with contrast hardener, which did not react with the given polymer. The observation of the microstructure of the samples and their porosity was realized four weeks after placing them in an aggressive environment. The microstructure of the sample sets labelled CIPP VES2 and CIPP VES3 was observed. The microstructure of the CIPP samples exposed to an aggressive environment was also observed to better explain the changes of the flexural properties of CIPPs when exposed to an aggressive environment.

## 3. Results and Discussion

### 3.1. How Flexural Properties and AE Are Affected by an Aggressive Environment

#### 3.1.1. Comparison of Flexural Properties and Cumulative Hits

**Figure 4:**
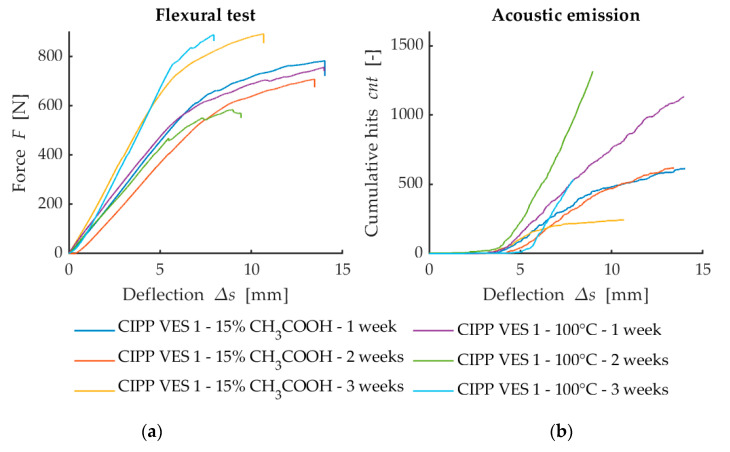
Comparison of the flexural loading and AE results after exposure to chemical aggressive media (15% CH_3_COOH) and thermal loading in dryer (100 °C): (**a**) Dependence of flexural force on deflection; (**b**) Dependence of cumulative hits (cnt) on deflection.

**Figure 5:**
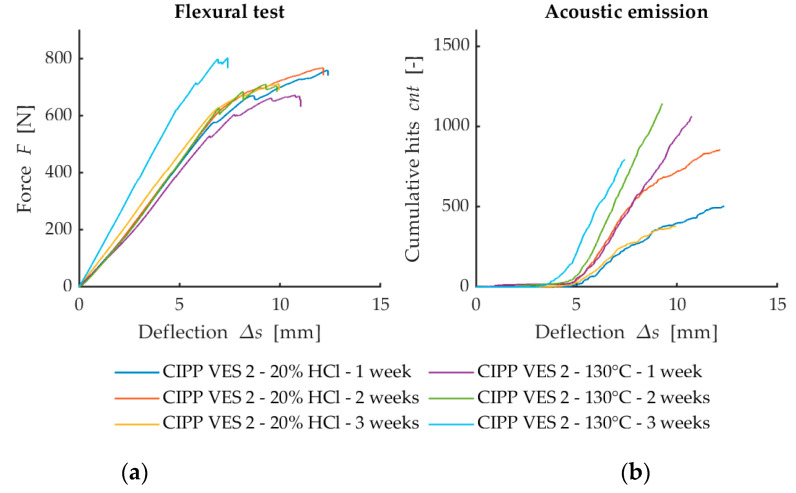
Comparison of the results from the flexural loading and AE after exposure to chemical aggressive media (20% HCl) and thermal loading (130 °C): (**a**) Dependence of flexural force on deflection; (**b**) Dependence of cumulative hits (cnt) on deflection.

**Figure 6:**
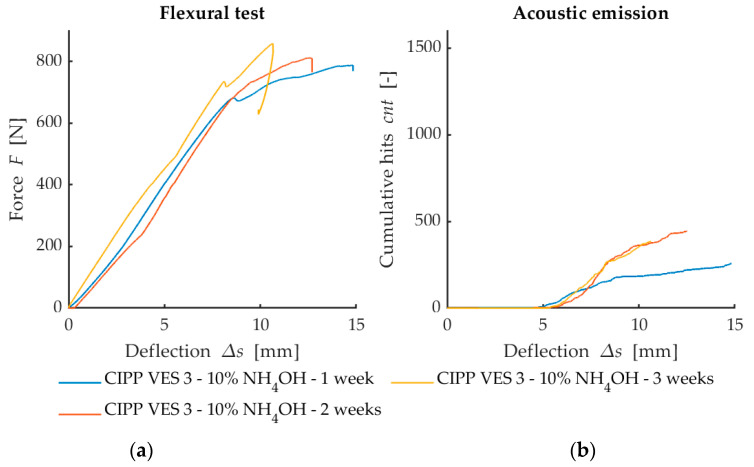
Comparison of the results from flexural loading and AE after exposure to chemical aggressive media (10% NH_4_OH): (**a**) Dependence of flexural force on deflection; (**b**) Dependence of cumulative hits (cnt) on deflection.

**Figure 7:**
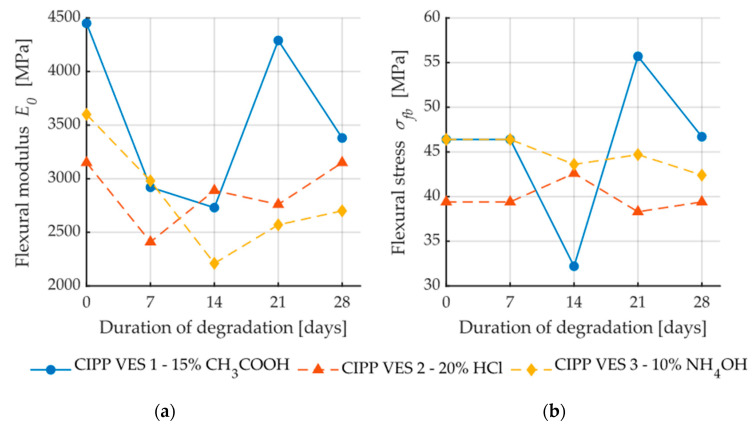
Dependence of flexural properties on the time of exposure to aggressive media: (**a**) Short-term flexural modulus; **(b)** Flexural stress.

The flexural force deflection curves from flexural loading are provided in Figure 4a, Figure 5a and Figure 6a. They show a dependency between the flexural force acting on the sample and deflection of the sample. The first point where the linear development of loading is changed (force decreases when deflection increases) represents failure of the sample. This value is used for the calculation of flexural strength at break. Figure 7 illustrates that after the CIPP VES3 sample was exposed to NH_4_OH for three weeks, its flexural properties were also improved. This positive effect can also be observed on the samples submerged in 15% CH_3_COOH. This can be explained by the crystallization of the individual solutions in the open surface porous structure of the samples (the surface in contact with the host pipe), which would improve the stiffness of the samples. However, as Figure 7 shows, the corrosive environment of 20% HCl caused the flexural properties of the CIPP samples to worsen, both in terms of flexural strength and short-term flexural modulus. Generally, VES resins offer excellent resistance to acids and alkalis, and high-density cross-linked products are suitable for temperatures above 120 °C. The opposite trend of the set of samples CIPP VES 2 compared to the samples VES 1 and VES 3 is probably due to the different porosity of the tested CIPP VES 2 samples; the difference in porosity was 8%. This porosity difference could result in the HCl solution penetrating to different depths of the internal structure of the samples, which may affect the flexural properties of the CIPP samples. Furthermore, this difference compared to samples exposed to the solutions of 15% CH_3_COOH and 10% NH_4_OH may be due to a different solution density and viscosity, which could cause the different rate of solution penetration into the samples.

**Figure 8:**
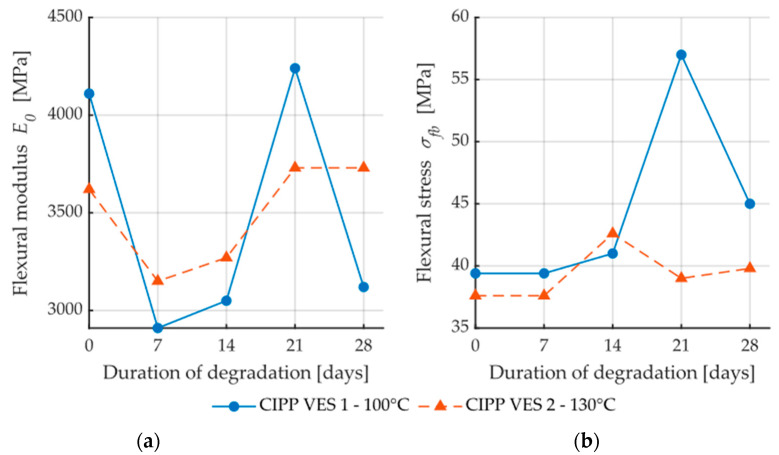
Dependence of flexural properties on the time of exposure to higher temperature: (**a**) Short-term flexural modulus; (**b**) Flexural stress.

Samples CIPP VES1 and CIPP VES2 showed improvement of flexural properties after being placed in a dryer at temperatures of 100 °C and 130 °C for three weeks (Figure 8). It can be assumed that the samples’ polymerization was improved by the high temperatures enhancing the VES network of the bonds. Only sample CIPP VES2 showed a decrease in flexural strength at break after being exposed to an increased temperature (130 °C) for three weeks; however, its flexural elasticity modulus was not decreased. Therefore, it can be stated that for the purpose of enhancing the flexural properties of VES-based CIPP samples, a temperature of approximately 100 °C should be applied for three weeks.

The development of the dependency of flexural force on deflection (Figure 4a) implies that the exposure of CIPP to a 15% solution of CH_3_COOH or a thermal load in a dryer at 100 °C for two weeks causes an increase of toughness compared to exposure for only one week. When the exposure time was prolonged to three weeks, it was observed that the CIPP VES1 samples became brittle. The results from the AE testing, which show that after two weeks of exposure, the samples had a higher cumulative number of AE overshoots when subjected to the same influence compared to the samples that had been exposed for three weeks, complement these mechanical properties (Figure 4b). A comparison of both degradation environments did not reveal any considerable differences regarding the mechanical properties. However, when it comes to AEs, slight differences could be observed, in particular, the increased AE activity of the samples exposed to a temperature of 100 °C. This could indicate lower structural damage than that observed after exposure to a 15% solution of CH_3_COOH.

The development of curves of loading force depending on deflection (Figure 5a) implies that exposure to a 20% solution of HCl or a temperature of 130°C for one week does not cause a considerable difference in the mechanical behavior of sample set CIPP VES2. A considerable difference can be observed between the cumulative number of overshoots of AE and deflection (Figure 5b), with the sample degraded by exposure to hydrochloric acid (20% HCl) having a considerably lower number of overshoots than the sample thermally loaded in a dryer at 130 °C. This difference in behavior is probably caused by larger structural damage of the sample from being in 20% HCl, with the structural bonds being damaged, and, consequently, no new failures that would show AE activity were created. The only difference in behavior can be observed on the samples placed in 20% HCl for two weeks. This different behavior was probably caused by the formation/deposition of crystals in the material’s structure, which would be a source of AE signals during a flexural test.

The development of loading force curves depending on deflection (Figure 6a) implies that the material showed strengthening after exposure to 10% NH_4_OH. This is apparent from the curves in Figure 6a, with the highest force being required to break the sample after three weeks of degradation. However, the AE activity (Figure 6b) did not provide a simple conclusion. This was mainly because the sample exposed to an aggressive environment for one week attained the lowest AE value. From a purely AE point of view, it can be concluded that the structure of this sample was mostly deteriorated by the action of the degrading solution; however, it could also be caused by the higher porosity of the sample. This hypothesis was not confirmed by the increased activity of the sample, which was degraded for three weeks. The increased activity of this three-week sample could also be caused by the formation/deposition of new crystals in the structure of the observed material, which generates a higher resistance to breakage.

The results shown in Figure 4, Figure 5 and Figure 6 suggest that the simultaneous measurement of AE during loading tests on specimens with three-point flexure is advantageous. The curve does not make clear the influence that degradation has on the test samples. However, the record of AE activity shows differences between the individual degraded test samples [33]. For this reason, it is a suitable indicator of the level of deterioration of the internal structure of test samples being subjected to mechanical load.

**Figure 9:**
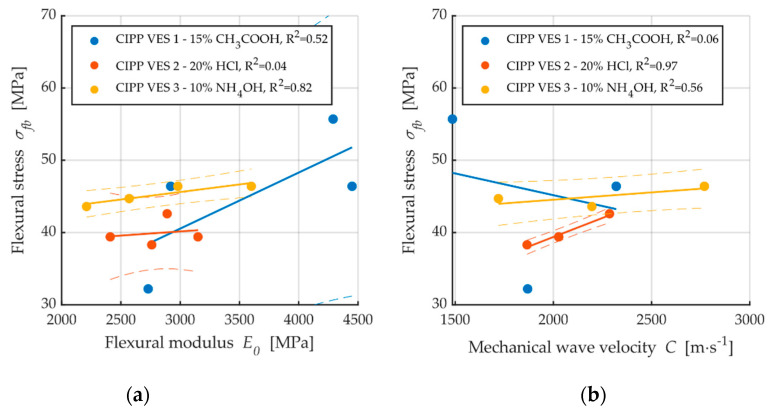
Dependencies of flexural properties after exposure to chemical aggressive media: (**a**) Dependence of flexural stress to short-term flexural modulus; (**b**) Dependence of flexural stress to mechanic wave velocity.

**Figure 10:**
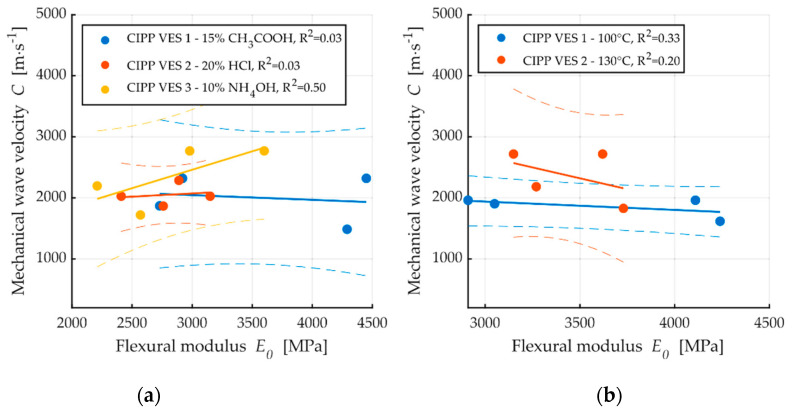
Dependence of short-term flexural modulus on mechanic wave velocity: (**a**) After exposure to chemical aggressive media; (**b**) After exposure to high temperatures.

Figure 9a shows dependence between flexural stress and flexural modulus; a correlation between these two properties is apparent. The coefficient of correlation is only low for the samples exposed to the aggressive environment of 20% HCl (R^2^ = 0.04); this is caused by the fact that, after two weeks, both the elasticity modulus and flexural strength at break values increased, and then subsequently decreased. Conversely, Figure 9b demonstrates a contradictory phenomenon where the dependence of flexural strength and mechanic wave velocity for the samples CIPP VES1 have nearly no correlation (R^2^ = 0.06); however, the samples exposed to the liquid acidic environment of 20% HCl show an apparent correlation between flexural stress and mechanic wave velocity (R^2^ = 0.97). These results imply that the dependence of individual tested parameters—both the flexural properties of CIPP samples and AE parameters—depend on the type of aggressive environment. Figure 10 shows the dependence of short-term flexural modulus on mechanic wave velocity. The samples exposed a temperature of 100 °C exhibited higher correlation than those exposed to a temperature of 130 °C (Figure 10b). Dependence between short-term flexural modulus and mechanic wave velocity was not proven for samples exposed to an aggressive environment.

#### 3.1.2. Velocity of Propagation of Acoustic Wave in Material

**Figure 11:**
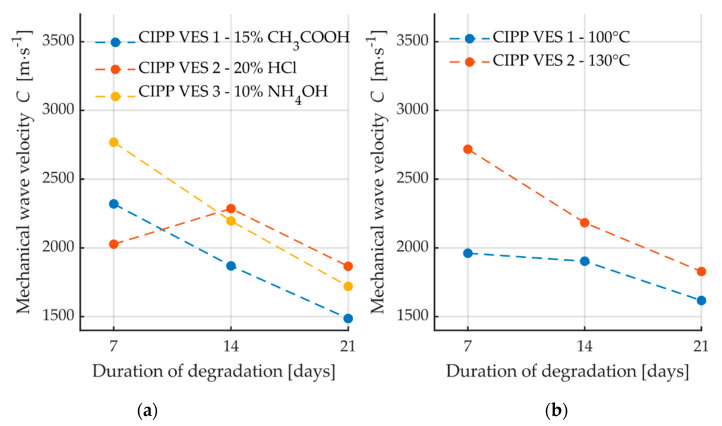
Dependence of mechanical wave velocity on the duration of CIPP sample degradation: (**a**) After exposure to chemical aggressive media; (**b**) After exposure to high temperatures.

For a more complex image of the structure of the observed degraded materials, the velocities of the propagation of the waves in the materials were measured before the destructive flexural test. A comparison of the velocities of the generated c waver (with Pen-Test) in the materials is provided in Figure 11.

A comparison of the individual velocities showed a reduction of velocity after the prolonged action of the degrading environment. This decrease of velocity was probably caused by the disturbance of the inner structure of the material, i.e., the formation of obstacles to wave propagation. The only exception was the sample that was placed in 20% HCl for two weeks, whose velocity increased. This confirms the assumption of the formation of crystalline phase as this facilitates the easier propagation of mechanical waves in the material. The observed reduction of velocity was probably caused by disturbance of the internal structure of the material, i.e., the formation of obstacles to wave propagation.

**Figure 12:**
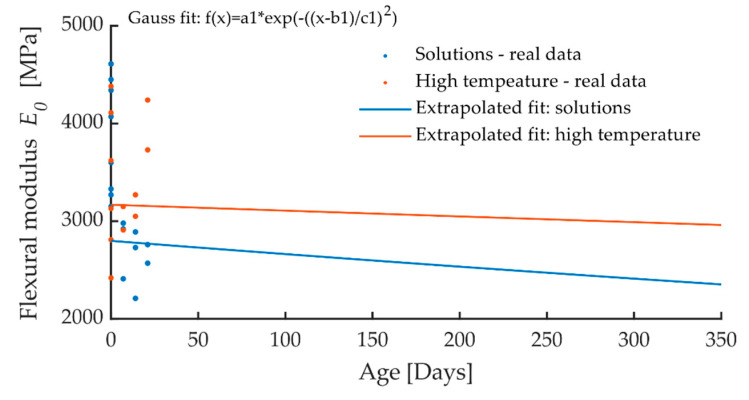
Extrapolation for prediction of the change in the modulus of elasticity of the CIPP samples loaded by the aggressive environment (chemical aggressive media and thermal loading) after one year.

To predict the change of CIPP flexural properties after exposure to a chemical aggressive environment and high temperature, extrapolation using a Gaussian model for a short-term flexural modulus was calculated using the Matlab software. It is obvious from Figure 12 that chemical aggressive media exert a greater effect on the flexural modulus than high temperatures. Based on the extrapolation, it can be assumed that after one year of CIPP exposure to aggressive chemical solutions, there will be an approximately 15% reduction in flexural properties, while exposure to high temperatures will probably result in only a 5% reduction.

### 3.2. Microstructure

**Figure 13:**
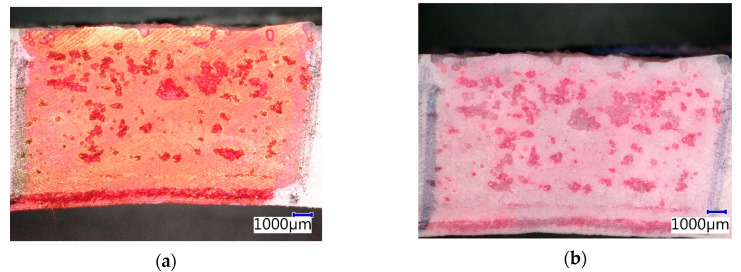
Photomicrographs of CIPP VES1 under the optical microscope: (**a**) Before chemical stress; (**b**) After four weeks’ immersion in 15% CH_3_COOH.

After four weeks of exposure to a 15% solution of acetic acid, the CIPP VES1 sample displayed a change in the color of contrasting agent (Figure 13). The optical digital microscope image shows the irregular and uneven surface of the sample (upper edge). The sample was taken from a place that did not adhere to the host pipe. If a CIPP pipe adheres closely to a host pipe, the outer surface of the former conforms to the surface of the host pipe. Hence, if the surface of the host pipe is smooth, the outer surface of the CIPP pipe is also smooth. The samples were hardened in the earthenware host pipes usin, which explains the higher proportion of open air pores. It can also be observed that, even after four weeks in 15% acetic acid, there was no significant degradation of the internal structure of the sample or inner membrane, and no separation of the individual layers of saturated and hardened fabric was observed. About 2 mm from the lower edge, the network in the layer of the composite is visible (Figure 13). The porosity of the CIPP VES1 samples was around 10%, which is apparent in the cross section in Figure 13.

**Figure 14:**
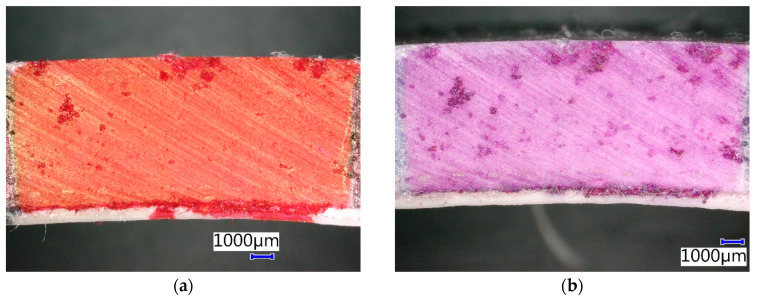
Photomicrographs of CIPP VES2 under the optical microscope: (**a**) Before chemical stress; (**b**) After four weeks’ immersion in 20% HCl.

Figure 14 shows that there was also a change of contrasting agent color in the CIPP VES2 sample after four weeks of immersion in 20% HCI. No additional changes of the microstructure of this CIPP sample were observed. It can be assumed that the surface of the sample was impregnated with the 20% HCl solution and it got into the open pores of the sample. Generally, it is known that VES-based CIPPs have excellent resistance to inorganic acids, which was also proven by this experiment. Its flexural properties were even enhanced after two weeks of exposure to 20% HCl.

**Figure 15:**
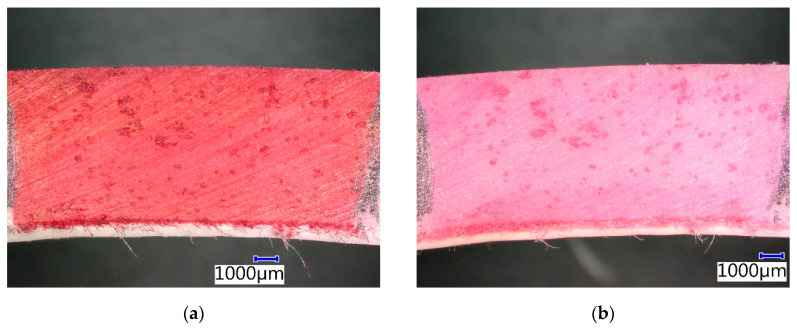
Photomicrographs of CIPP VES3 under the optical microscope: (**a**) Before chemical stress; (**b**) After four weeks immersion in 10% NH_4_OH.

CIPP VES3 only showed a change of color of the contrasting agent, specifically, a lightening, after four weeks of exposure to a 10% solution of ammonia hydroxide (Figure 15). The external surface of the sample was smooth, unlike that of CIPP VES1 which was exposed to a 15% solution of acetic acid. This is related to the minimal number of open pores in the sample through which the aggressive medium can access the CIPP. These samples showed better flexural properties after three weeks in a 10% solution of NH_4_OH. Figure 15 shows that no degradation occurred. It can be assumed that the open pores were filled with the solution, which then crystallized, thereby increasing the elasticity modulus of the CIPP sample.

**Figure 16:**
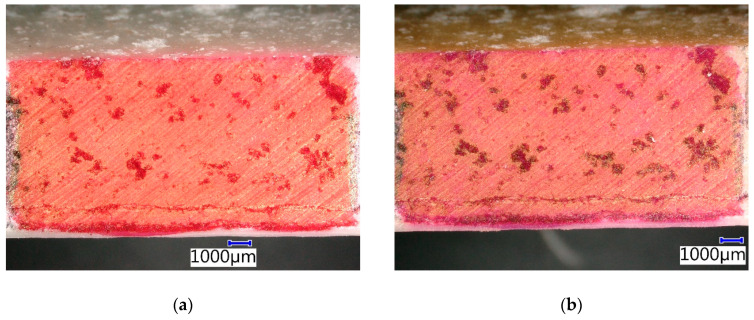
Photomicrographs of CIPP VES1 under the optical microscope: (**a**) Before thermal stress in a dryer; (**b**) After four weeks of thermal stress in a dryer at a temperature of 100 °C.

The effect of increased temperature on sample CIPP VES1 manifested mainly in a color change of the contrasting agent, which made the air pores more visible (Figure 16). The amount of pores was approximately 10%. No further manifestation of material degradation was observed, i.e., neither in the bearing system nor in the inner membranes. The external surface of the CIPP sample, which was in contact with the host pipe made from earthenware, changed color; however, this had no negative effect on the properties of the CIPP sample. The joint between the internal and middle layer of the CIPP sample was apparent on the bottom edge of the sample, approximately 1 mm from the inner membrane. The testing of its flexural properties implied that the CIPP sample finished polymerization when exposed to a temperature of 100 °C, which is related to the improvement in its mechanical properties (Figure 9)—the macromolecules of the VES created an additional network.

**Figure 17:**
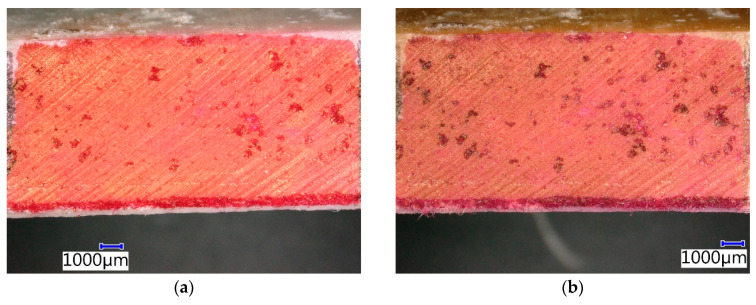
Photomicrographs of CIPP VES2 under the optical microscope: (**a**) Before thermal stress in a dryer; (**b**) After four weeks’ thermal stress in a dryer at a temperature of 130 °C.

The air pores of sample CIPP VES2, which were exposed to 130 °C, encompassed approximately 6% of the examined surface. A temperature of 130 °C caused a change in the of color of the contrasting agent and a slight change of the resin system. However, the images in Figure 17 and the flexural properties results (Figure 8) imply that neither the microstructure of the CIPP samples, nor their mechanical properties, were diminished. For this reason, it can be stated that the samples of VES-based CIPP examined are capable of long-term resistance to increased temperatures, specifically 130 °C. The only effect is a change in color of the outer layer of the sample, which would not be exposed to the aggressive environment in real life, as it would be in direct contact with the renovated pipe.

## 4. Conclusions

This paper researched and described the effect of chemical and thermal stress on the short-term mechanical properties of CIPP saturated with VES by means of AE changes of activity of the structure of CIPP samples during three-point flexural loading. It was found that AE is a more sensitive means of evaluation of the influence of degradation processes on the structure of test samples than stress-deflection, which is a commonly used method. The results of the AE measurements imply that some changes that took place within the material structure during degradation processes could not be detected via a three-point flexural test. In particular, the AE activity of more degraded test samples was lower because of the highly deteriorated structure of the material; at the same time, the composite material was more ductile (flexible), which implies that the deformation of the sample increased, depending on the intensity of the flexural force compared to the reference samples. Considerable enhancement of the flexural properties of CIPP was obvious after exposure of CIPP to increased temperatures, particularly 100 °C. Our results imply that the renovation CIPP pipes saturated with VES can resist specific aggressive environments and thermal stress. Using an optical microscope, it was found that no internal failures that would have a negative effect on the long-term durability of CIPP in an aggressive environment had occurred.

## Figures and Tables

**Table 1:** Geometrical dimensions of the samples used for the determination of flexural properties.

Sample	Aggressive Environment	Duration	Width [mm]	Thickness ^1^ [mm]	Length [mm]	Radius of Curvature R2 [mm]
CIPP VES1	15% CH_3_COOH	1 week	49.94	7.71	148.15	146.14
		2 weeks	49.99	7.62	148.19	146.18
		3 weeks	49.96	7.83	148.21	146.12
		4 weeks	49.98	7.74	148.17	146.15
CIPP VES2	20% HCl	1 week	49.97	8.81	158.14	145.58
		2 weeks	49.95	9.02	158.03	145.48
		3 weeks	49.96	9.08	157.98	145.43
		4 weeks	49.95	9.05	158.02	145.69
CIPP VES3	10% NH_4_OH	1 week	50.03	8.60	153.80	145.69
		2 weeks	50.07	9.02	154.04	145.52
		3 weeks	49.96	9.13	153.87	145.42
		4 weeks	50.00	9.11	154.14	145.61
CIPP VES1	100 °C	1 week	49.97	7.72	7.70	146.13
		2 weeks	49.98	7.62	7.63	146.15
		3 weeks	49.98	7.92	7.84	146.18
		4 weeks	49.96	7.78	7.75	146.05
CIPP VES2	130 °C	1 week	49.95	8.61	8.61	145.69
		2 weeks	49.98	8.92	8.83	145.57
		3 weeks	49.91	9.14	8.91	145.40
		4 weeks	49.93	9.17	8.85	145.53

^1^ Thickness of samples includes EPDM inner layer.
